# Dystrophic Scoliosis in Neurofibromatosis and Rib-head Resection: A Case Report

**DOI:** 10.5704/MOJ.1711.007

**Published:** 2017-11

**Authors:** KL Chong, KS Lam, Z Zuki

**Affiliations:** Department of Orthopaedics, Hospital Sungai Buloh, Sungai Buloh, Malaysia; ^*^AOSpine Centre, London Bridge Hospital, London, United Kingdom

**Keywords:** neurofibromatosis scoliosis, dystrophic curve, rib-head excision

## Abstract

Surgical management of scoliosis in Neurofibromatosis type I may be challenging at times especially when dealing with dystrophic curves. We highlight the importance of meticulous study of the radiological imaging and careful pre-operative planning in a patient with dystrophic scoliosis.

## Introduction

Scoliosis in neurofibromatosis (NF) can present as a simple straight forward scoliosis or complicated dystrophic scoliosis^1^. Dystrophic scoliosis requires detailed study of the radiological images to identify the presence of dystrophic features because severe dystrophic changes are associated with faster deterioration of the curve progression and also to prevent neurological complications peri-operatively^2,3^. Early surgical stabilization is indicated in patients with high risk factors for curve progression^3,4^. Rib-head dislocation and penetration into spinal canal at the apical curve is not uncommon which leads to the debate as to whether it should be resected to prevent neurological deficit^4,5^.

## Case Report

The patient was a fit and active 15-year old boy with no known family history of neurofibromatosis. He had noticed some deformity in his spine when he was 6 years old, and was diagnosed with neurofibromatosis type I (NF-1) and followed up by a neurologist until the age of 9 years. Physical examination when he presented to us at our Spine Centre revealed no neurological deficit other than hyper-reflexia in both lower limbs. The abdominal reflexes were intact. There was minor limb length discrepancy but patient was able to ambulate without any problem. The patient stood with level shoulders and level pelvis. He also had a khyphotic deformity of the spine which became more pronounced during forward Adam’s test.

**Fig. 1:**
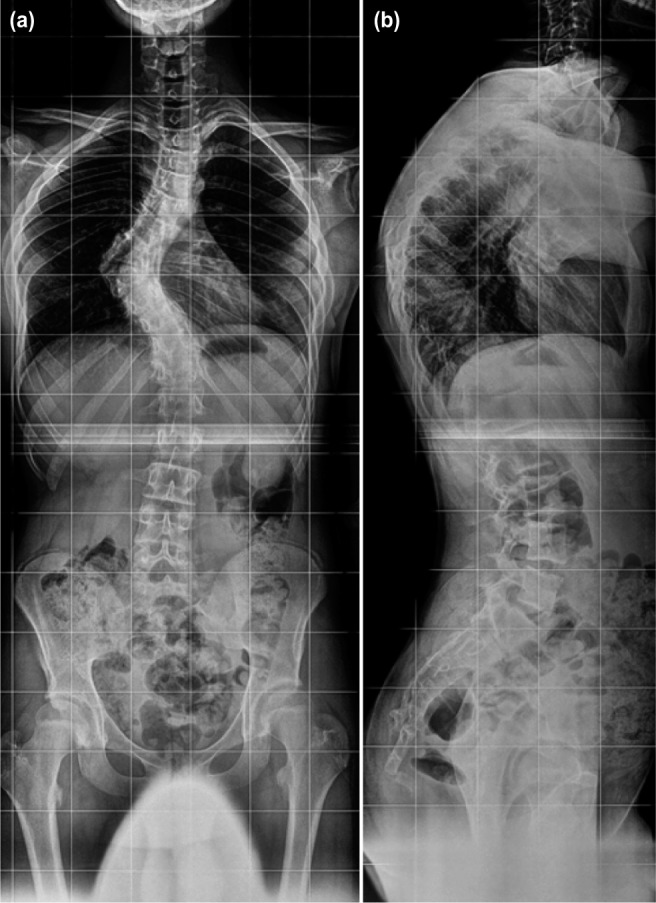
Standing whole spine radiographs showing Cobb angles of (a) sharp angular right-sided 64 degrees upper thoracic scoliosis (T5-T9), and (b) 74 degrees kyphosis (T4-T11).

During his initial follow-up with the neurologist, serial plain radiographs that were performed every two years had revealed mild progression of the scoliosis. However, the curve rapidly progressed as he entered the adolescent growth spurt. When the patient presented himself at our Spine Centre, he was Risser 3, and the radiological imaging showed a classical upper thoracic kyphoscoliosis with Cobb angles measuring 64 degrees scoliosis (T5-T9), and 74 degrees kyphosis (T4-T11) but without any evidence of severe NF-1 features such as rib-pencilling (Fig. 1). Extension bolster radiographs and right anteroposterior (AP) side bending views showed more than 50% correction of his deformity suggestive of a relatively flexible curve.

**Fig. 2:**
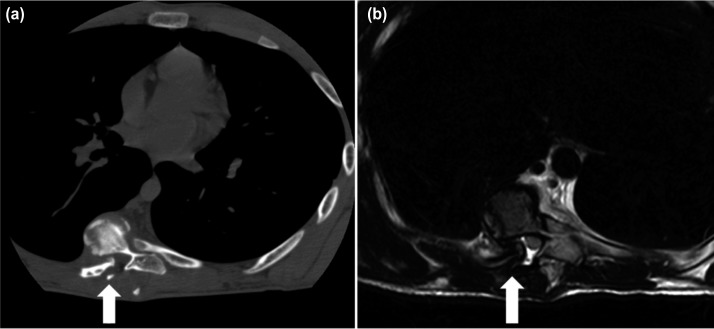
Axial cut imaging showing a dislocated right-sided T8 rib head into the spinal canal; (a) CT scan, and (b) T2-weighed MRI where the dura is indented but with no obvious cord compression (see arrows).

Computed tomography (CT) scan showed the tip of T8 rib-head had dislocated dorsally into the spinal canal. Magnetic resonance imaging (MRI) showed no intraspinal anomaly such dural ectasia, cord compression or myelomalacia changes although the spinal canal appeared to be very narrow at the apex centred at T7/T8 (Fig. 2). There was a neurofibroma tumour in the concavity of the scoliosis at the level of T4 measuring approximately 2cm in diameter.

**Fig. 3:**
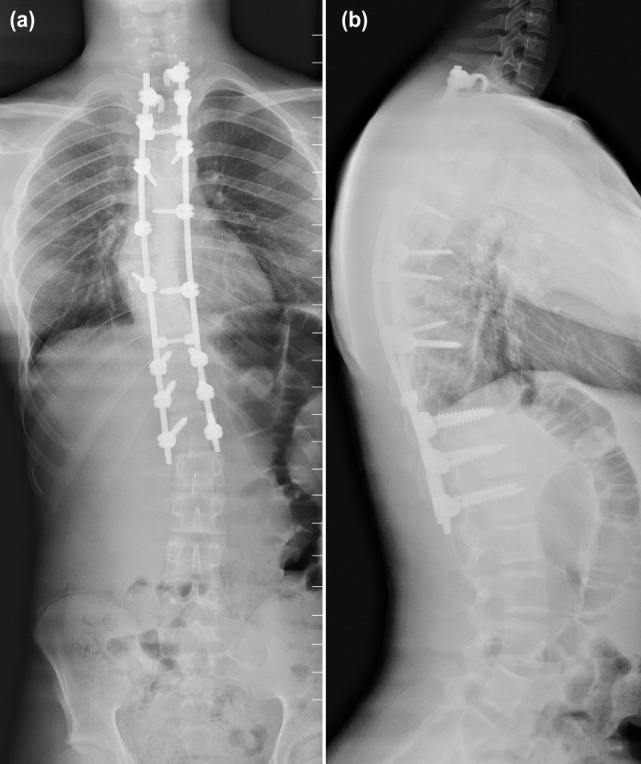
Post-operative whole spine standing; (a) AP and (b) lateral views showing excellent correction of the thoracic kypho-scoliosis.

The patient was previously treated in a TLSO brace for 12 months which he had worn consistently during that period. He also had undergone shock wave therapy that had resulted in a patch of redness over the kyphotic area. This was probably the cause of the oedema that was seen on the MRI scan rather than implicating an abnormal area NF tissue. The patient underwent surgery on 20 August 2016. Posterior instrumentation was performed from T1 to L1 using a combination of segmental pedicle screws distally and supralaminar hooks proximally into T1 to T2 (Fig. 3). Smith-Peterson osteotomies were performed at all instrumented levels and the interspinous ligaments from T1/T2 until T12/L1 were removed. Prior to the correction of the curve, hemi-laminectomies were performed at T7 and T8 followed by resection of the right dislocated T8 rib-head which was located at the convex side of the apex. After the curve correction using a cantilever technique followed by derotation and apical convex rod manoeuvre corticotomies were performed followed by application of local bone autograft and allograft. The overall surgery was uneventful. Post-operatively, patient recovered well without any neurological deficit. The brisk lower limb reflexes that were present prior to the surgery had disappeared immediately after surgery.

## Discussion

Neurofibromatosis is an autosomal dominant chromosomal disorder characterized by scoliosis, café-au-lait spots, pseudarthrosis of long bones and limb overgrowth. Neurofibromatosis can be further divided into neurofibromatosis-1 (NF-1) and neurofibromatosis-2 (NF-2). The most common skeletal presentation of neurofibromatosis is scoliosis, with the incidence ranging from 10-60%^1^.

This patient had a typical presentation of neurofibromatosis characterized by short segment kyphoscoliotic deformity of the proximal thoracic spine, with short curve with severe apical rotation from T4 to T9. The clinical presentation and radiological imaging in this patient that were very suggestive of dystrophic features included a) early occurrence at the young age of 6 years, b) presence of neurofibroma lesion at T4 spine, c) dislocated T8 rib-head into the spinal canal, d) acute angular scoliotic deformity, and e) sagittal plane kyphoscoliosis. Absent dystrophic features in this patient were vertebral scalloping, pencilling of the ribs or transverse processes, foraminal enlargement and defective pedicles. The more severe the dystrophic changes, the faster is the progression of the curve^1,2^.

Early surgical stabilization was indicated in this patient because of the risk factors such as substantial progression of the curve at an early age of onset, skeletal immaturity, a high Cobb angle during initial presentation, kyphosis of more than 50 degrees and location of the apex of the curve in the middle to caudal of thoracic area. However, the patient and his parents had opted for non-surgical treatment in the initial stages with brace. Therefore, it was not surprising that bracing had failed, and with the rapid curve progression, surgical intervention was deemed necessary in the end^3,4^.

A careful and thorough physical examination and assessment of all radiological images is mandatory when planning for surgery in dystrophic scoliosis. This is to minimize any intra and peri-operative complications. Features of vertebral or intraspinal lesion, dural ectasia, spinal cord pseudomeningocele, protrusion of the neurofibroma through neural foramina, spinal cord compression over a sharp angular kyphosis and impingement of rib on spinal cord through the foramina need to be identified as they can cause catastrophic neurologic compromise after surgical correction. It has been reported that the incidence of rib-head dislocation and penetration into spinal canal at the apical curve was about 15.9%^5^. In this case, the usage of CT-scan and three-dimensional reconstruction have been instrumental in visualizing the complex bony anatomy, extent of deformity, size of pedicles and most importantly, identifying the presence of a dislocated rib-head.

MRI of whole spine is indicated for dystrophic scoliosis and it is important to determine the internal content of the spinal canal, presence of space occupying lesion and about the spinal cord itself. It was fortunate that the dislocated T8 rib-head into the spinal canal was detected both by the CT-scan and MRI prior to surgery, thereby enabling us to plan ahead for resection of the rib-head prior to correction of the deformity. Otherwise, the rib-head could easily compromise the spinal cord during correction of the deformity especially during derotation and lengthening of the curve. The process of excising the rib-head was fairly easy and quick, taking no more than ten minutes with minimal blood loss. The process involved a hemi-laminectomy at the level of dislocated rib-head followed by the use of pituitary rongeurs to remove it once it was clearly visualized. Once the rib-head has been resected, the correction of the deformity can be done without worrying about compression of the spinal cord^2-4^.

In this case, the use of supralaminar hooks at T1 and T2 were necessary because of the small diameter pedicle at these levels and also to prevent implant pull out. There was no evidence of lamina thinning or presence of dural ectasia or neurofibroma within the spinal canal.

A generous amount of bone graft using a mixture of both biological and autologous bone graft from the morselized spinous processes and laminae was applied to the decorticated lamina, especially around the apex of the curve. Plenty of bone graft is necessary in NF-1 scoliosis as the incidence of pseudoarthrosis is very high. This is due to the fact that bone grafts surrounded by abnormal neurofibromatous tissue have an increased tendency to resorb in the midportion due to its highly vascular nature. Evaluation of the fusion mass should be routinely performed at six months to identify any evidence pseudoarthrosis. If there is any radiological evidence of weakness of the fusion mass, re-exploration and augmentation of the fusion should be undertaken^4,5^.

In conclusion, management of NF-1 scoliosis involves careful pre-operative study of radiological imaging to look for evidence of dystrophic features to minimize neurological complications during and after surgery. Dystrophic feature such as rib-head dislocation must be identified and the surgical plan should include a hemi-laminectomy at the affected levels followed by rib-head resection prior to correction of the curve. Although the surgical method used in this case has been widely discussed and reported previously, there is no consensus when it comes to rib-head resection. Some authors had suggested leaving it alone while others had resected the rib-head only if it was found at surgery to be too close to or compressing the spinal cord intraoperatively^1,5^. However, we would like to recommend routine resection of the rib-head(s) at the apex of the dystrophic curve regardless of any evidence of cord compression because it is a simple, quick and straight forward procedure with minimal blood loss, thus eliminating the high risk of a catastrophic neurological sequelae secondary to acute spinal cord injury.
